# Functional characterization and hormonal regulation of the *PHEOPHYTINASE* gene *LpPPH* controlling leaf senescence in perennial ryegrass

**DOI:** 10.1093/jxb/erv509

**Published:** 2015-12-06

**Authors:** Jing Zhang, Guohui Yu, Wuwu Wen, Xiqing Ma, Bin Xu, Bingru Huang

**Affiliations:** ^1^College of Agro-grassland Science, Nanjing Agricultural University, Nanjing, 210095, PR China; ^2^Department of Plant Biology and Pathology, Rutgers, the State University of New Jersey, New Brunswick, NJ 08901, USA; Received 27 August 2015; Revised and Accepted 6 November 2015

**Keywords:** ABA, Chl degradation, cytokinin, ethylene, leaf senescence, PPH.

## Abstract

Chlorophyll (Chl) degradation occurs naturally during leaf maturation and senescence, and can be induced by stresses, both processes involving the regulation of plant hormones. The objective of this study was to determine the functional roles and hormonal regulation of a gene encoding pheophytin pheophorbide hydrolyase (PPH) that catabolizes Chl degradation during leaf senescence in perennial grass species. A PPH gene, *LpPPH*, was cloned from perennial ryegrass (*Lolium perenne* L.). LpPPH was localized in the chloroplast. Overexpressing *LpPPH* accelerated Chl degradation in wild tobacco, and rescued the stay-green phenotype of the Arabidopsis *pph* null mutant. The expression level of *LpPPH* was positively related to the extent of leaf senescence. Exogenous application of abscisic acid (ABA) and ethephon (an ethylene-releasing agent) accelerated the decline in Chl content in leaves of perennial ryegrass, whereas cytokinin (CK) and aminoethoxyvinylglycine (AVG; an ethylene biosynthesis inhibitor) treatments suppressed leaf senescence, corresponding to the up- or down-regulation of *LpPPH* expression. The promoters of five orthologous *PPH* genes were predicted to share conserved *cis*-elements potentially recognized by transcription factors in the ABA and CK pathways. Taken together, the results suggested that *LpPPH*-mediated Chl breakdown could be regulated positively by ABA and ethylene, and negatively by CK, and *LpPPH* could be a direct downstream target gene of transcription factors in the ABA and CK signaling pathways.

## Highlight

Leaf senescence regulated by abscisic acid, cytokinins, and ethyelene was associated with transcriptional regulation of *LpPPH*. Orthologous *PPH* genes share conserved *cis*-elements potentially targeted by hormonal signaling pathways.

## Introduction

Leaf senescence is a highly regulated natural process during the life of a leaf, which involves degradation of various macromolecules, such as chlorophyll (Chl) and proteins (Breeze *et al.*, 2011; Fukao *et al.*, 2012). Premature leaf senescence leads to demolished photosynthesis, retarded plant growth and productivity (Guo and Gan, 2014). Chl degradation is the common hallmark of leaf senescence. Chl degradation involves multiple biochemical reactions catalyzed by at least six Chl catabolic enzymes (CCEs) in the chloroplast, namely pheophytin pheophorbide hydrolyase (PPH), Non-Yellow Coloring1 (NYC1), NYC1-like (NOL), Chl *a* reductase (HCAR), pheide *a* oxidase (PAO), and red Chl catabolite reductase (RCCR) (Hörtensteiner, 2006; Barry, 2009; Hörtensteiner and Kräutler, 2011). PPH, also named NYC3 in rice (*Oryza sativa*) or CRN1 in Arabidopsis, is one of the key CCEs, which catalyzes conversion of pheophytin *a* to pheophorbide *a* by cleaving off the phytol group (Schelbert *et al.*, 2009). The *PPH* transcript level is positively correlated with the extent of leaf senescence during the natural leaf maturation process (Schelbert *et al.*, 2009) or is induced by stresses, such as dark or shade (Wei *et al.*, 2011; Liu and Guo, 2013). However, the regulatory mechanisms of *PPH* expression associated with natural or stress-induced leaf senescence are not well understood.

Plant hormones, such as abscisic acid (ABA), ethylene, and cytokinin (CK), are known to play roles in regulating both natural leaf senescence and that induced by stress (Thomas and Stoddart, 1980; Grbić and Bleecker, 1995; Yang *et al.*, 2002; Dodd and Beveridge, 2006; Lim *et al.*, 2007; Kim *et al.*, 2015). Recently, a number of independent studies suggested a possible link between Chl catabolism and ABA, ethylene, and CK signaling pathways (Büchert *et al.*, 2011; Nakjima et al., 2012; Delmas *et al.*, 2013; Zwack and Rashotte, 2013; Cheng and Guan, 2014; Koyama, 2014; Qiu *et al.*, 2015). ABA at high concentrations may accelerate leaf senescence and Chl degradation (Thomas and Stoddart, 1980). Büchert et al. (2011) and Cheng and Guan (2014) found that exogenous spraying of ethylene also induced the Chl degradation associated with higher transcript levels of Chl catabolism genes in both pear and broccoli. In the model plant Arabidopsis, ethylene-insensitive mutants (*etr1*, *ein2*/*ore2*, and *ein3*) had a slower rate of Chl degradation during leaf senescence (Oh *et al.*, 1997; Sakai *et al.*, 1998; Li *et al.*, 2013; Qiu *et al.*, 2015). Furthermore, EIN3, together with ORE1/NAC2, transcription factors in the ethylene signaling pathway, promotes Chl degradation by enhancing the promoter activity of Chl degradation genes (such as *NYE1* or *SGR*, *NYC1*, and *PAO*) (Qiu *et al.*, 2015). In contrast to ABA and ethylene, CK is known to be negatively correlated with the level of leaf senescence (Zwack and Rashotte, 2013). The correlation between a decreased endogenous CK level in leaves and the onset and progression of senescence has been documented (Tao *et al.*, 1983). The increase of endogenous CK content through overexpression of the CK synthesis gene, *isopentenyl-transferases* (*IPT*), could significantly extend the lifespan of leaves in various plant species (Li *et al.*, 1992; Gan and Amasino, 1995; Merewitz *et al.*, 2010). Despite the importance of the involvement of ABA, ethylene, and CK in leaf senescence, it is unclear whether ABA-, ethylene-, or CK- mediated leaf senescence is related to the regulation of *PPH* genes for Chl breakdown during leaf senescence.

Leaf senescence is a major problem for perennial grasses which are harvested for their green foliage as forage or maintained for a uniform green canopy as is the case for turfgrasses. Knowledge of mechanisms controlling leaf senescence is particularly important for developing stay-green grasses and improving forage or turf quality. As discussed earlier, *PPH* is a key gene involved in Chl degradation, but how this gene is regulated during leaf senescence in plants, particularly in perennial grass species, is still unclear. Manipulating the expression of *PPH* through genetic modification or mutagenesis could lead to a stay-green phenotype in plants (Morita *et al.*, 2009; Schelbert *et al.*, 2009). In this study, we hypothesized that *PPH* cloned from a perennial grass species could possess the conserved functionality in Chl degradation, and PPH-mediated leaf senescence could be regulated by ABA, ethylene, or CK. The objectives of this study were (i) to determine the functional roles of *PPH* in leaf senescence in a perennial grass species (*Lolium perenne* L.) widely used as forage and turfgrass; (ii) to identify the regulatory roles of ABA, ethylene, and CK in *PPH* expression; and (iii) to predict the potential transcriptional factors activating or suppressing *PPH* expression conserved across diverse plant species.

## Materials and methods

### Plant materials and growth conditions

Perennial ryegrass (cv. ‘Pinnacle’) plants were used for cloning of the *PPH* gene and evaluation of *PPH* expression in relation to leaf senescence. Plants were grown in plastic pots (20cm in diameter and 25cm in height) filled with the mixture of soil and peat (3:1 v/v) and maintained in growth chambers (Environmental Growth Chamber, Chagrin Falls, OH, USA) set at 25/20 °C (day/night), 70% relative humidity, and 12h photoperiod with photosynthetically active radiation (PAR) of 750 µmol photons m^–2^ s^–1^.

The function of the *PPH* gene from perennial ryegrass in leaf senescence was confirmed using transient transformation and mutant complementation assay in two model plant species, wild tobacco (*Nicotiana benthamiana*) and Arabidopsis. Wild tobacco plants were cultured under the same conditions as for perennial ryegrass.

*Arabidopsis thaliana* wild-type (Col-0) and T-DNA insertion lines (*at5g13800-1*, SALK_000095) were grown in 10cm^2^ pots with peat soil, and were maintained in growth chambers (Environmental Growth Chamber) set at 22 °C with a 16h photoperiod and a PAR of 350 µmol photons m^–2^ s^–1^. The T-DNA line was obtained from the Arabidopsis Biological Resource Center (ABRC) (Alonso *et al.*, 2003). All plants were well watered, and fertilized weekly with half-strength Hoagland’s nutrient solution (Hoagland and Arnon, 1950).

### Evaluation of leaf senescence in perennial ryegrass induced by darkness and affected by ABA, ethephon, AVG, and CK

Leaf senescence affected by ethephon, aminoethoxyvinylglycine (AVG), ABA, and CK and induced by darkness was evaluated in perennial ryegrass. For dark-induced leaf senescence, the fully expanded leaves (~12 d after leaf emergence) were excised from perennial ryegrass plants, and were kept in between paper towels moistened with 3mM MES buffer (pH 5.8) at 25 °C for 8 d in the dark. For ethephon, AVG, ABA, and CK treatment, the protocol reported by Sato *et al.* (2009) was used. The fully expanded leaves (~12 d after leaf emergence) were excised from perennial ryegrass plants, and incubated in 3mM MES buffer (pH 5.8) supplemented with water (control), 200 µM ethephon, 25 µM AVG, 50 µM ABA, or 25 µM 6-benzylaminopurine (6-BA) at 25 °C.

Several physiological parameters commonly used as indicators of leaf senescence were evaluated, namely leaf Chl content, photochemical efficiency, and membrane stability (Liu and Guo, 2013; Tian *et al.*, 2013; Yamatani *et al.*, 2013). Chl was extracted by soaking leaves in dimethylsulfoxide (DMSO) for 48h and the absorbance of extracts was measured at 663nm and 645nm using a spectrophotometer (Spectronic in Instruments, Rochester, NY, USA) (Barnes *et al.*, 1992). Leaf photochemical efficiency was expressed as the ratio of the variable fluorescence (*F*_v_) to the maximal fluorescence (*F*_m_) (*F*_v_/*F*_m_) (Oxborough and Baker, 1997). Chl fluorescence was determined using a fluorescence meter (Dynamax, Houston, TX, USA) after leaves were dark adapted for 30min. Leaf membrane stability was estimated by electrolyte leakage (EL) from leaves (Murray *et al.*, 1989). For EL measurement, 0.2g of leaves were collected, washed three times, and then immersed in 30ml of deionized water. Initial conductivity (C_i_) was measured with a conductivity meter (YSI Model 32, Yellow Spring, OH, USA) after shaking overnight. Leaves were then boiled in an autoclave for 20min for measurement of the conductance (C_max_). The leaf relative EL was calculated as 100×C_i_/C_max_.

### Isolation and sequence analysis of *PPH* from perennial ryegrass (*LpPPH*)

Total RNA was extracted from senescent leaf samples of perennial ryegrass. A pair of primers (*LpPPH*-F and *LpPPH*-R; Supplementary Table S1 available at *JXB* online) was designed for cloning the conserved nucleotide sequence of *LpPPH* according to the alignment of the *PPH* genes in rice and brachypodium. Two gene-specific primers (*LpPPH*-3' RACE and *LpPPH*-5' RACE; Supplementary Table S1) were designed to amplify the ends of the *PPH* gene. The cDNA used for random amplification of cDNA ends (RACE)-PCR was synthesized with a SMARTer^®^ RACE 5'/3' Kit (Clontech Laboratories, Mountain View, CA, USA) following the manufacturer’s instructions. PCRs were performed in a 50 µl reaction volume using Q5 High-Fidelity 2× Master Mix (New England Biolabs, Ipswich, MA, USA). The DNA fragments obtained from the PCRs were sequenced and aligned to obtain the full-length open reading frame (ORF) of *PPH*. The ratio between non-synonymous and synonymous nucleotide substitutions (Ka/Ks) was calculated using DNAsp5 software (http://www.ub.edu/dnasp/) (Librado and Rozas, 2009) for selected pairs of homologous genes. The *cis*-element analysis of selected *PPH* promoters (–2000bp from the transcription initiation site) was performed using the PLACE website (http://www.dna.affrc.go.jp/PLACE/) (Higo *et al.*, 1999).

### Plasmid construction

The ORFs of *LpPPH* and *AtPPH* were amplified with gene-specific primers (LpPPH-CDSF, LpPPH-CDSR, AtPPH-CDSF, and AtPPH-CDSR), listed in Supplementary Table S1 at *JXB* online, using Q5 High-Fidelity 2× Master Mix (New England Biolabs), cloned into pENTR/D, their sequences were confirmed, and then they were moved into the destination vector pEarleyGate103 (Earley *et al.*, 2006) for overexpression analysis or into a modified Gateway-compatible p2GWF7.0 vector (Karimi *et al.*, 2005) for subcellular localization analysis.

### Subcellular localization of LpPPH

Arabidopsis mesophyll protoplasts were isolated from mature leaves of 6-week-old plants following the procedure described in Endler *et al.* (2006). The 3'-green fluorescent protein (GFP)-tagged LpPPH was transformed into protoplasts using 20% polyethylene glycol, then incubated in the dark at 22 °C for 36h, and examined under a Zeiss LSM 780 laser scanning confocal microscope (Carl Zeiss SAS, Jena, Germany). GFP fluorescence images were viewed at an excitation wavelength of 488nm, and the emission signal was recovered between 495nm and 530nm according to published procedures (Schelbert *et al.*, 2009). Chl autofluorescence was viewed between 643nm and 730nm.

### Transient overexpression of *LpPPH* in wild tobacco

Mature leaves of 4- to 6-week-old wild-type tobacco plants were used for the experiment applying the method described previously (Yang *et al.*, 2001). Briefly, *Agrobacterium* strain *AGL1* harboring pEarleyGate103 with or without the target gene was injected into a tobacco leaf at the concentration of OD_600_=0.6. After injection, the tobacco leaves were excised and incubated on wet filter paper at 25 °C in the dark for up to 6 d. This experiment was repeated three times, each time with at least three biological replicates.

### Arabidopsis mutant complementation assay

The homozygosity of the *pph* mutant of Arabidopsis was confirmed with three sets of primers, given in Supplementary Table S1 at *JXB* online. The standard floral dip method was applied for transformation (Clough and Bent, 1998). Putative transgenic lines were selected on Murashige and Skoog (MS) medium with 20mg l^–1^ glufosinate, and were further verified by PCR. For the Arabidopsis senescence assay, detached leaves (numbers 4–6) of 28-day-old plants were excised and incubated on wet filter paper at 22 °C in total darkness for up to 6 d.

### Analysis of gene expression with real-time RT-PCR

Total RNAs were extracted using an RNA cleaning kit (Qiagen, Valencia, CA, USA) following the manufacturer’s instructions. After DNA digestion with TURB DNA-free™ (Life Technologies, Grand Island, NY, USA), first-strand cDNA was synthesized using a High Capacity cDNA Reverse Transcription kit (Applied Biosystems, Grand Island, NY, USA). For semi-quantitative RT-PCR, the reaction was performed in a 20 μl reaction volume using PCR super mix (Life Technologies) for 26 cycles. Primers used are listed in Supplementary Table S1 at *JXB* online, with *ACTIN2* as the reference gene.

For qRT-PCR, the reaction was performed in a 20 μl reaction volume with power SYBR^®^ Green PCR Master mix (Applied Biosystems) using Roche Light Cycler^®^ 480 II Real-Time PCR Systems. All reactions were performed with two technical and three biological replicates. Primers used for qRT-PCR are listed in Supplementary Table S1 *. eIHF4A* and *TEF1* were used as reference genes (Huang *et al.*, 2014).

### Statistical analysis

Data in this study were statistically analyzed using one-way ANOVA, and the means were compared by the LSD and Duncan test at a significance level of 0.05 by using SPSS (version 12, SPSS Inc., Chicago, IL, USA). The data are expressed as means ± SE.

## Results

### RACE-PCR cloning and phylogenetic analysis of *LpPPH*

**Fig. 1.**
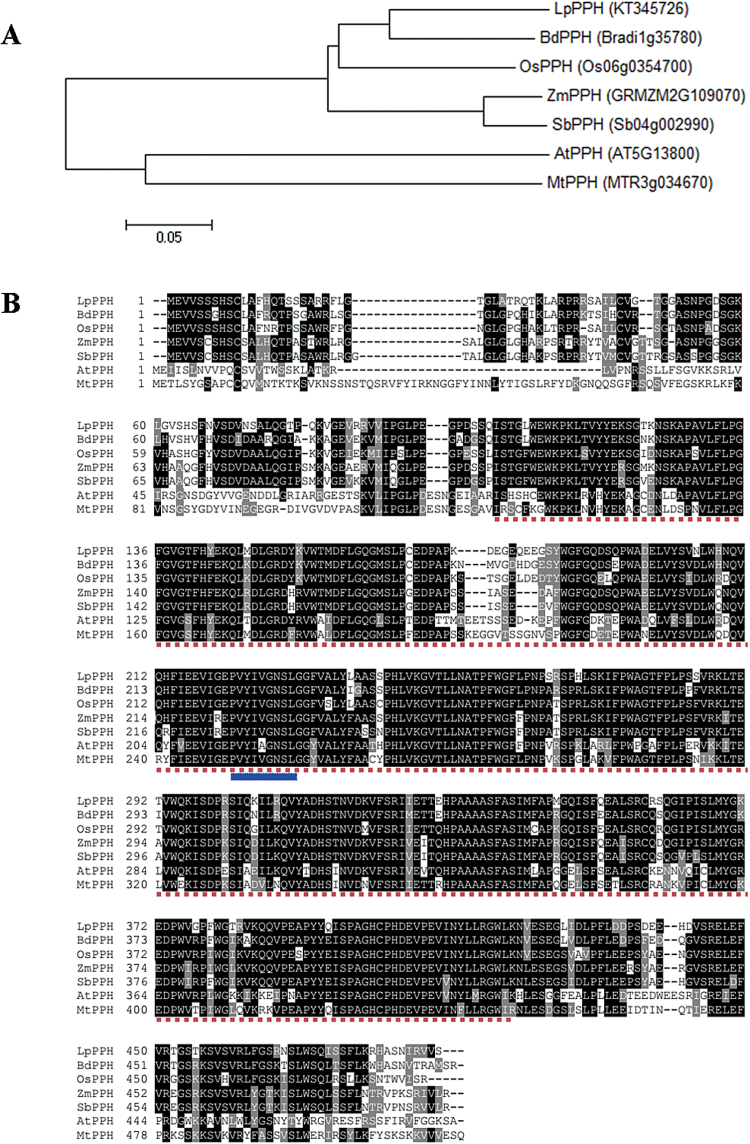
Phylogenetic analysis and multiple sequence alignment of seven PPHs. (A) Phylogenetic analysis: the Neighbor–Joining phylogenetic tree was constructed using MEGA6 with the sum of branch length=1.16285194. In addition to LpPPH, its orthologous proteins in Arabidopsis (AtPPH), rice (OsPPH), brachypodium (BdPPH), maize (ZmPPH), sorghum (SbPPH), and *Medicago truncatula* (MtPPH) were used for the analysis. (B) Multiple sequence alignment: note that the region under dotted lines is the conserved domain of α/β hydrolase-fold family proteins, and the sequence under solid lines (VYIV/AGNSL) is the PPH motif (Schelbert *et al.*, 2009). (This figure is available in colour at *JXB* online.)

According to the nucleotide sequence alignment between *OsPPH* and *BdPPH*, a pair of primers was designed to amplify a conserved sequence fragment of *PPH* from the cDNA derived from senescent leaves of ryegrass. The full-length coding sequnce (CDS) of *LpPPH* (GenBank accession no. KT345726), obtained using 5'- and 3'-RACE-PCR, encodes a deduced protein of 488 amino acids, with a pI of 6.55 (ExPASy server, http://web.expasy.org/computepi/) and the conserved domain of α/β hydrolases (InterPro IPR029058; PLN02824) (Schelbert *et al.*, 2009) (Fig. 1).

A phylogenetic tree of LpPPH and six PPH orthologs of different plant species was constructed (Fig. 1A), in which the phylogenetic relationship between the PPHs was consistent with that of the plant species, indicating that the cloned *LpPPH* should be the functional pheophytinase-encoding gene. The cloned *LpPPH* shared 87.9, 82.0, and 62.1% nucleotide sequence identity with *BdPPH*, *OsPPH*, and *AtPPH*, respectively. Based on the phylogenetic relationship of the PPH orthologs, the synonymous and non-synonymous nucleotide substitution rates were further compared between the closely related pairs, demonstrating that all *PPH* genes were under purifying selection (Ka/Ks <1) (Table 1).

**Table 1.** Purifying selection of *PPH* genes

****Ortholog 1****	****Ortholog 2****	****Ks****	****Ka****	****Ka/Ks****	****Evolutionary selection****
*****LpPPH*****	*****BdPPH*****	0.3179	0.0836	0.263	Purifying
*****BdPPH*****	*OsPPH*	0. 6018	0.1059	0.176	Purifying
*****OsPPH*****	*ZmPPH*	0.8235	0.1099	0.133	Purifying
*****ZmPPH*****	*SbPPH*	0.2066	0.0388	0.188	Purifying
*****AtPPH*****	*MtPPH*	1.6381	0.2537	0.155	Purifying

Ks, number of synonymous substitutions per synonymous site; Ka, number of non-synonymous substitutions per non-synonymous site.

When Ka/Ks=1, neutral evolution; Ka/Ks <1, purifying selection; Ka/Ks >1, diversifying selection.

### LpPPH localized in chloroplasts

**Fig. 2.**
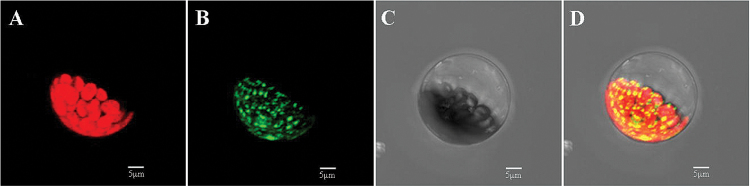
Subcellular localization of LpPPH. (A) Chlorophyll autofluorescence; (B) green fluorescence from LpPPH–GFP; (C) bright field; (D) merge of green and autoflorescence.

LpPPH protein was predicted to have a 72 amino acid chloroplast transit peptide at the N-terminus without a significant transmembrane domain using the ChloroP1.1 (Emanuelsson *et al.*, 1999), pSORT (Horton *et al.*, 2007), and TMHMM servers, respectively (the prediction result of pSORT is presented in Table 2). Transient overexpression of LpPPH fused with a GFP tag at the C-terminus in Arabidopsis mesophyll protoplasts confirmed that LpPPH was localized in chloroplasts (Fig. 2). Because LpPPH was predicted to contain no transmembrane domain, it may be mainly localized in the chloroplast stroma.

**Table 2.** Subcellular localization predicted by pSORT (Horton *et al.*, 2007)

Predicated subcellular location	Affirmativity
Chloroplast stroma	0.883
Microbody (peroxisome)	0.64
Chloroplast thylakoid membrane	0.577
Chloroplast thylakoid space	0.53

### Overexpression of *LpPPH* induced leaf senescence in wild tobacco and complemented the Arabidopsis *pph* mutant phenotype

**Fig. 3.**
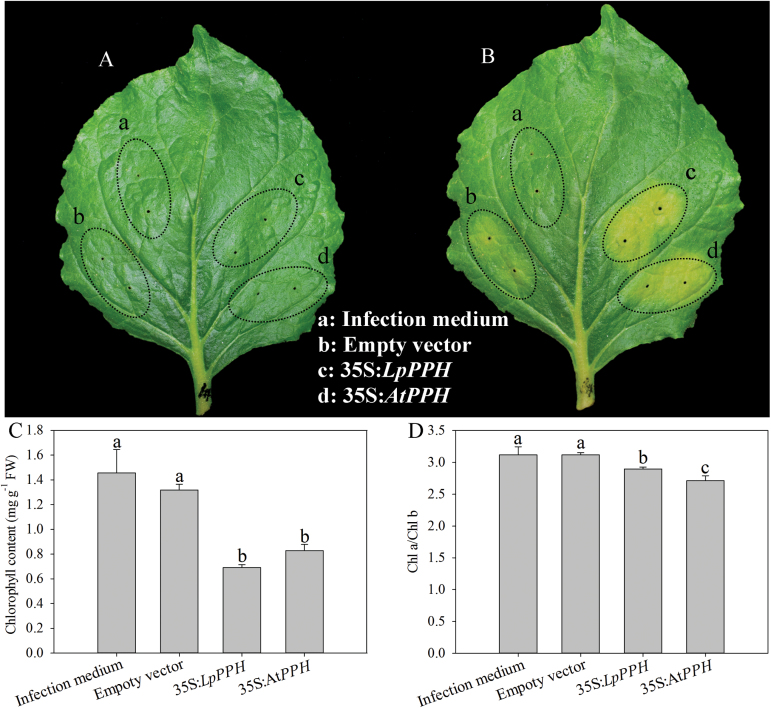
Transient overexpression of *LpPPH* in wild tobacco (*N. benthamiana*). *Agrobacterium* strain AGL1 harboring either empty vector or *LpPPH*/*AtPPH* genes was injected into mature leaves of wild tobacco. (A) 0 day after infection; (B) 6 d after infection; (C) Chl content 6 d after infection; (D) Chl *a*/Chl *b* ratio 6 d after infection. Data are means ±SE (n=*4* in C and D).

In order to confirm the function of *LpPPH* in regulating leaf senescence, the transient overexpression of this gene in wild tobacco and a complementation assay of the Arabidopsis *pph* mutant were performed using both *LpPPH* and *AtPPH* driven under control of the *Cauliflower mosaic virus* (CaMV) 35S promoter. The transient overexpression of 35S:*LpPPH* and 35S:*AtPPH* both accelerated leaf senescence and resulted in a significantly lower Chl content and Chl *a*/Chl *b* ratio than those of the controls (Fig. 3).

**Fig. 4.**
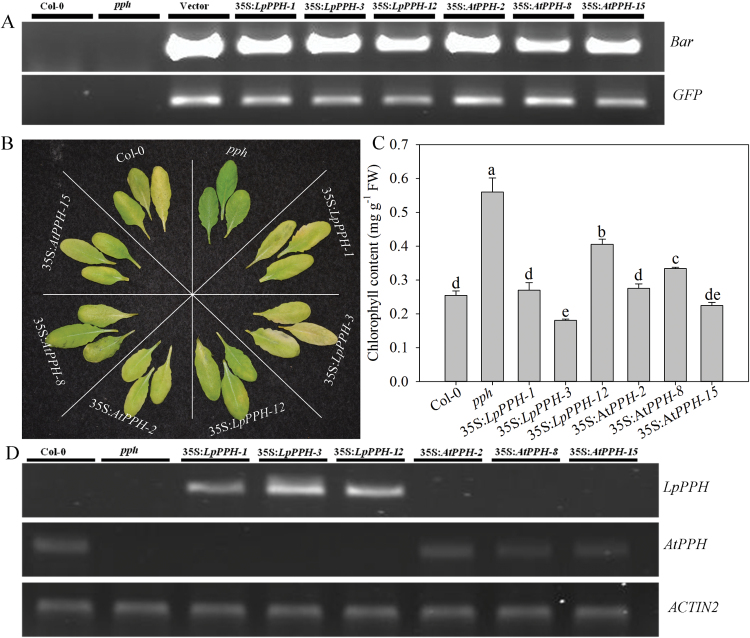
*LpPPH* complements the leaf’s stay-green phenotype of the Arabidopsis *pph* mutant. (A) PCR confirmation of the presence of *Bar* and *GFP* genes in transgenic lines. (B) 35S:*LpPPH* as well as 35S:*AtPPH* complemented the phenotype of the *pph* mutant. The photograph was taken 6 d after dark-induced senescence on detached leaves. (C) Chl content of the detached leaves of each line 6 d after dark-induced senescence. (D) RT-PCR analysis of the expression levels of *LpPPH* or *AtPPH* in the tested Arabidopsis lines using detached leaves 6 d after dark-induced senescence. Data are means ±SE (*n*=4 in C and D).

**Fig. 5.**
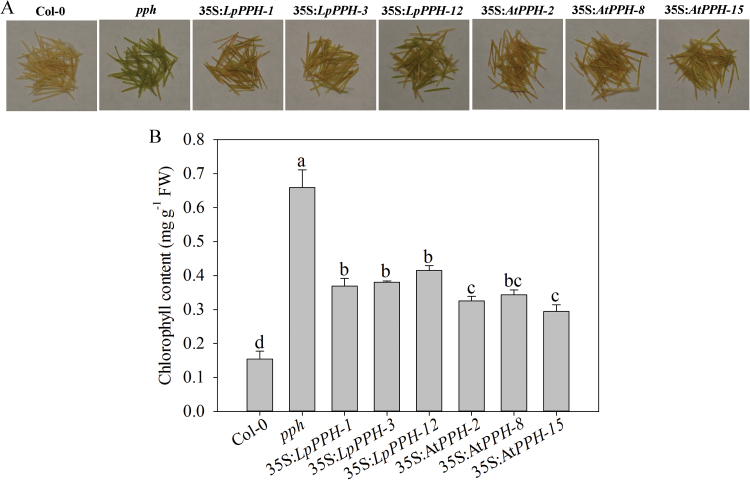
*LpPPH* under the control of the CaMV 35S promoter partially restored the seedpod’s stay-green phenotype of the Arabidopsis *pph* mutant. (A) Phenotype of the wild type, *pph* mutant, and transgenic lines. (B) Chl content of the tested lines. Data are means ±SE (*n*=4 in C).

For *pph* mutant complementation assay, three T_2_ homologous transgenic lines of each gene (***35S*:** *LpPPH*/*pph-1*, *-3*, and *-12*, and 35S:*AtPPH*/*pph-2*, *-8*, and *-15*) were compared together with the wild type and *pph* controls (Fig. 4). After 6 d of dark treatment, the detached leaves of the Arabidopsis *pph* mutant showed the stay-green phenotype, while Col-0 and all transgenic lines exhibited severe leaf Chl degradation (Fig. 4). Moreover, the expression levels of *LpPPH* and *AtPPH* were negatively correlated with Chl content in leaves of each transgenic line (Fig. 4C, D). In addition, *LpPPH* also rescued the stay-green phenotype of the seedpods of the Arabidopsis *pph* mutant (Fig. 5). Taken together, these results demonstrated that *LpPPH* complemented the phenotype of the Arabidopsis *pph* null mutant and shared the same functionality with *AtPPH*.

### Increasing transcript levels of *LpPPH* in senescent leaves

**Fig. 6.**
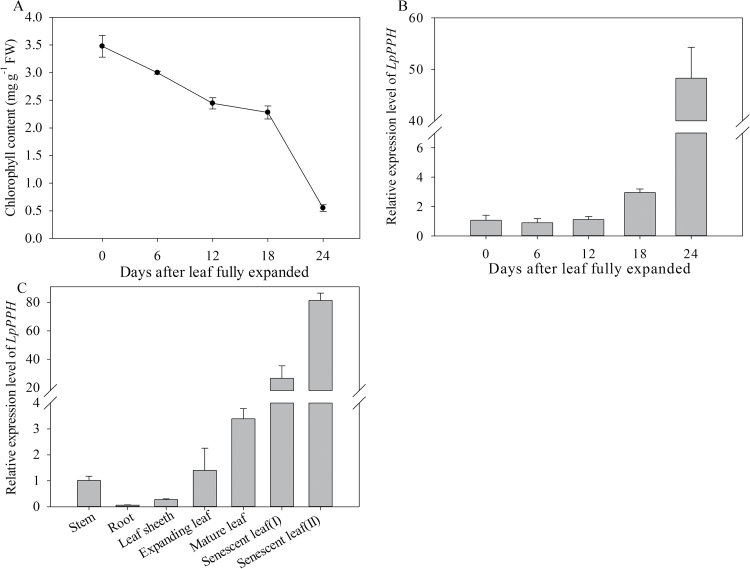
Expression levels of *LpPPH* in leaves during senescence and in different organs. (A) Chl content changes in fully expanded leaves (note that perennial ryegrass leaves were fully expanded 12 d after leaf emergence). (B) Changes in relative expression of *LpPPH* in fully expanded leaves. (C) Relative expression of *LpPPH* in different organs. Data are means ±SE (*n*=4).

**Fig. 7.**
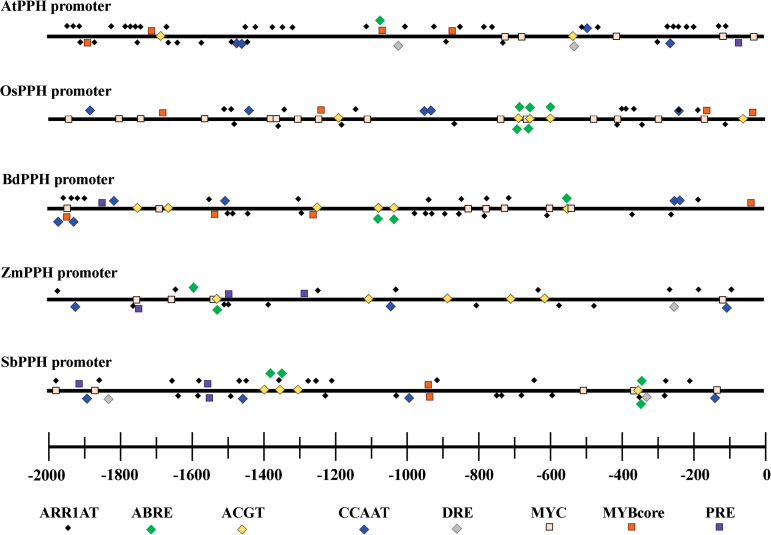
List of conserved *cis*-elements involved in CK and ABA pathways and in response to abiotic stresses in five *PPH* promoters.

Our previous test showed that the leaf of the tested ryegrass variety reached its full length and highest Chl content at 12 d after leaf emergence, and Chl content declined thereafter (data not shown), indicating that those leaves reached full maturation at 12 d after emergence. Therefore, in this study, changes in Chl content and *LpPPH* transcript level were examined during leaf senescence using the 12-day-old mature leaves (referred to as mature leaf) as the starting material (Fig. 6A). The transcript level of *LpPPH* did not significantly change during the early phase of leaf senescence (Chl declined by 32% from 3.4mg g^–1^ FW to 2.3mg g^–1^ FW), but significantly increased to the highest level when Chl decreased by 82% (from 3.4mg g^–1^ FW to 0.6mg g^–1^ FW) (Fig. 6B). The transcript level of *LpPPH* was also analyzed in different plant organs. The expression level of *LpPPH* was barely detectable in roots, and was relatively low in stems or leaf sheaths (Fig. 7C). The *LpPPH* gene had a ~30- and 80-fold higher expression level in leaves at early and late senescence stages (32 d and 36 d after leaf emergence), respectively, than in stems or in leaves at the expanding stage (6 d after leaf emergence) (Fig. 6C). 

### *LpPPH* is transcriptionally induced by ABA and ethephon, and suppressed by cytokinin and AVG

The analysis of promoters of *PPH* genes from five sequenced plant species demonstrated that all these *PPH* genes had multiple *cis*-elements involved in ABA or CK regulatory pathways. For example, ABRE (MACGYGB) and ACGT (G/AACGTC/T) are the core DNA-binding sequences for ABI3 and ABF4, respectively, and both transcription factors are in the ABA regulatory pathway. ARR1AT (NGATT) is the core sequence recognized and bound by Arabidopsis response regulators (ARRs), the transcription factors in the two-component CK signaling pathway (Fig. 7). In addition, a number of abiotic and biotic stress-responsive *cis*-elements were also found in all five *PPH* promoters, including a CCAAT box (heat shock element, CCAAT), a DRE (dehydration response element, RCCGAC), MYBCore (AtMYB1/2 DNA-binding site in response to water stress, CNGTTR), MYCATERD1 (MYC recognition sequence necessary for expression of erd1, and which can be bound by certain stress-inducible NAC proteins, CATGTG), and PRE (proline- and hypoosmolarity-responsive element, ACTCAT) (Fig. 7; Supplementary Table S2 at *JXB* online).

**Fig. 8.**
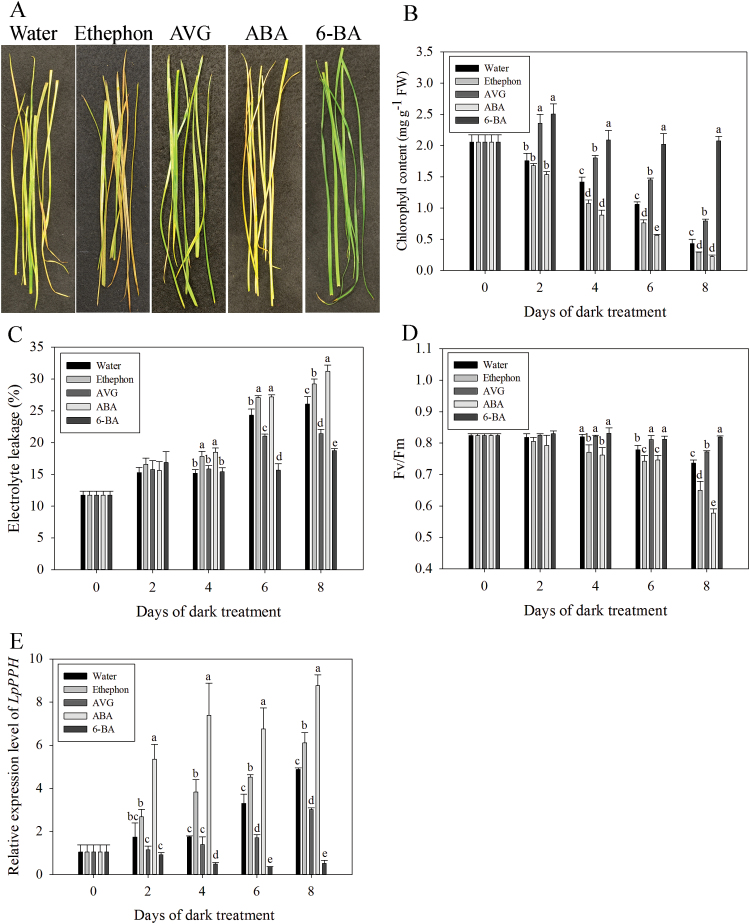
Effects of ABA, ethephon, AVG, and 6-BA on leaf senescence and on the relative expression of *LpPPH*. (A) Effects of ABA, ethephon, AVG, and 6-BA on detached leaves after 8 d in the dark. (B–E) Chl content (B), electrolyte leakage (C), *F*_v_/*F*_m_ (D), and relative expression of *LpPPH* changes after hormone treatment. Data are means ±SE (*n*=4 in A, B, and D, *n*=12 in C). (This figure is available in colour at *JXB* online.)

The test with exogenous treatment of leaves with ABA, ethephon, AVG, or 6-BA demonstrated that 6-BA and AVG significantly delayed leaf senescence, as manifested by increased Chl content and plasma membrane stability (or increased EL), and *F*_v_/*F*_m_; in contrast, ABA and ethephon treatment significantly accelerated leaf senescence, with leaves showing more a severe decline in Chl and *F*_v_/*F*_m_ but increased EL (Fig. 8). Consistent with the pattern of physiological changes during leaf senescence, 6-BA and AVG significantly suppressed the expression of *LpPPH*, but ABA and ethephon significantly induced its expression (Fig. 8).

## Discussion

A number of genes involved in the Chl degradation pathway were cloned from different plant species in recent years (review by Christ and Hörtensteiner, 2014). The *PPH* genes have been cloned from different plant species, such as Arabidopsis (Schelbert *et al.*, 2009; Ren *et al.*, 2010), rice (Morita *et al.*, 2009), and bamboo (*Bambusa emeiensis*) (Wei *et al.*, 2013), but not previously reported in perennial grass species. In this study, the *PPH* gene from perennial ryegrass was cloned and its function was confirmed. All PPHs have the conserved PPH motif (VYIA/VGNSL) (Schelbert *et al.*, 2009) in which the serine residue carries out the nucleophilic attack of the substrate for the hydrolytic enzymatic activity (Dodson and Wlodawer, 1998), although *LpPPH* only shared 87.9, 82.0, and 62.1% nucleotide sequence identities with *BdPPH*, *OsPPH*, and *AtPPH*, respectively. Transient expression of LpPPH fused to a GFP tag demonstrated that LpPPH was localized in the chloroplast, and the lack of a transmembrane domain in LpPPH further suggested that this protein may be mainly localized in the chloroplast stroma, which is consistent with the finding for AtPPH (Schelbert *et al.*, 2009). The synonymous and non-synonymous nucleotide substitution ratio (Ka/Ks) of <1.0 between the closely related *PPH* pairs demonstrated that *PPH* genes were under purifying selection (Librado and Rozas, 2009). Homologous characterization suggested that LpPPH from perennial ryegrass could share the conserved roles of PPH with other plant species in dephytylating Mg-free Chl during Chl catabolism.

The functionality of *LpPPH* was further confirmed experimentally with transient overexpression in wild tobacco and *pph* complementation assay in Arabidopsis. Overexpression of *LpPPH* resulted in leaf chlorosis in wild tobacco, while *LpPPH* rescued the stay-green phenotype in Arabidopsis *pph* null mutants. Furthermore, qPCR analysis demonstrated that the expression level of *LpPPH* was positively related to the greater extent of natural or dark-induced leaf senescence in perennial ryegrass, as manifested by the ~30- and 80-fold higher expression level in leaves at the early and late senescence stage in association with the decline in Chl content and increases in cell membrane stability. The association of increasing transcript levels of *OsPPH* with leaf senescence has also been reported in rice (Morita *et al.*, 2009). Our results strongly demonstrated that *LpPPH* served a function in Chl catabolism in perennial ryegrass, which could be manipulated to generate the stay-green trait that is desirable for perennial grass.

Further tests of *LpPPH* expression and physiological changes during leaf senescence in response to ethephon, AVG, ABA, and CK treatments showed that ethephon and ABA accelerated leaf senescence, and AVG and CK suppressed leaf senescence, which corresponded to up-regulated *LpPPH* and down-regulated *LpPPH*, respectively. This result supported that *PPH* is transcriptionally activated during leaf senescence and is regulated by sensing hormonal cues. The computational analysis of the *cis*-element components of five *PPH* genes showed that the *PPH* promoters shared conserved core nucleotide sequences potentially recognized by transcription factors in the ABA and CK signaling pathways, including ABRE, ACGT, and ARR1AT. ABRE and ACGT are the downstream core nucleotide sequences recognized by ABI3 and ABF4, respectively (Simpson *et al.*, 2003; Nakashima and Yamaguchi-Shinozaki, 2006), and both transcription factors are in the ABA regulatory pathway. The ACGT *cis*-element was also found in the promoter of another Chl degradation gene, *AtNYC1*, and was shown to be the DNA sequence targeted by ABF4 (Nakajima *et al.*, 2012). ARR1AT is the DNA recognition site for ARRs (Sakai *et al.*, 2000; Ross, 2004), which are transcription factors in the CK signaling pathway. These results suggested that *PPH* genes might be transcriptionally regulated by the ABA, ethylene, and CK pathway transcription factors. The identities of these transcription factors and their exact nucleotide binding will be experimentally screened and tested in the future. From the combined data of gene expression, phenotypic changes, and the analysis of *cis*-elements, we hypothesized that CK suppressed the expression of *PPH*, probably through the ARR transcription factors by recognizing some of the ARR1AT *cis*-elements, and thereby circumvented the progress of Chl degradation, whereas ABA induced the expression of *PPH*, probably either by directly recognition of the ABRE or ACGT *cis*-elements by ABI3 or ABF4 transcription factors in the ABA pathway or by indirectly recognizing the other *cis*-elements through downstream abiotic stress-inducible transcription factors (e.g. MYBs or NACs). The transcription factors involved in ethylene-induced leaf senescence and ethylene-activated *PPH* expression are as yet unknown. The recent report by Qiu *et al.* (2015) showed that most Chl catabolism genes but not *PPH* can be directly targeted by ORE1 in the ethylene signaling pathway. However, the fact that the promoter of the *LpPPH* gene lacked *cis*-elements in this study demonstrated that ethylene might not be directly involved in regulating the *LpPPH* gene through signaling and transcriptional activation. A number of *cis*-elements involved in abiotic stress were also found in the five PPH promoters in addition to ABA- or CK-responsive elements, including CCAAT box, DRE, MYBcore, MYCATERD1, and PRE. It is of interest that overexpressing DRE-binding proteins (DREBs) led to delayed instead of accelerated Chl breakdown under stressed growth conditions (Morran *et al.*, 2011; Bouaziz *et al.*, 2013). When and how the stress-inducible expression of transcription factors leads to accelerated or delayed Chl breakdown is still not well understood. Liang *et al.* (2014) found that a NAC protein, OsNAP, can directly bind to the promoter of *PPH* (*NYC3*). Yet the DNA-binding site of OsNAP has not yet been identified. The *cis*-element analysis suggested that the MYCATERD1 sequence that could be recognized by some stress-inducible NAC domain transcription factors (Tran *et al.*, 2004) should be a candidate DNA-binding site recognized by OsNAP. The presence of these conserved *cis*-elements provides us with a strong clue for identifying important transcription factors that may directly regulate Chl breakdown and for understanding the mechanism of abiotic stress-induced Chl degradation. It remains intriguing to discover the integral regulatory network of Chl catabolism upon the sensing of external and internal cues; for example, senescence-related hormones. Also the mechanisms of and binding specificities for ethylene-, ABA,- or CK-responsive transcription factors that may activate or deactivate *PPH* expression deserves further investigation.

### Conclusions

The *LpPPH* gene cloned from perennial ryegrass was confirmed to have a function in Chl phytol cleavage hydrolase, which could accelerate leaf senescence, depending on its expression levels, as demonstrated through the transient expression and mutant complementation assays, as well as qPCR analysis. LpPPH-mediated leaf senescence could be regulated by ethylene, ABA, and CK. Potential transcriptional factors in the ABA and CK signaling pathways were proposed as candidate upstream regulators of PPH that will be further confirmed by experimental approaches. The *LpPPH* gene could be used as a candidate gene by mutagenesis or gene editing techniques to create stay-green perennial grasses.

## Supplementary data

Supplementary data are available at *JXB* online.

Table S1. Primers used in this study

Table S2. Prediction of *PPH* promoters and *cis*-elements. 

Supplementary Data
