# Supplementary material for: Functional characterization and hormonal regulation of the PHEOPHYTINASE gene LpPPH controlling leaf senescence in perennial ryegrass
Source: J Exp Bot. 2015 Dec 6;67(3):935–45. doi: 10.1093/jxb/erv509 (PMC4737083; doi:10.1093/jxb/erv509)
Supplement: Supplementary Data [file supp_67_3_935__index.html]

Functional characterization and hormonal regulation of the PHEOPHYTINASE gene LpPPH controlling leaf senescence in perennial ryegrass — Functional characterization and hormonal regulation of the PHEOPHYTINASE gene LpPPH controlling leaf senescence in perennial ryegrass — Supplementary Data 

# Functional characterization and hormonal regulation of the *PHEOPHYTINASE* gene *LpPPH* controlling leaf senescence in perennial ryegrass

## Supplementary Data

Data files

- supplementary\_table\_S1.pdf - Supplementary Data
- supplementary\_table\_S2.xlsx - Supplementary Data
