# Supplementary material for: Functional characterization and hormonal regulation of the PHEOPHYTINASE gene LpPPH controlling leaf senescence in perennial ryegrass
Source: J Exp Bot. 2015 Dec 6;67(3):935–45. doi: 10.1093/jxb/erv509 (PMC4737083; doi:10.1093/jxb/erv509)
Supplement: Supplementary Data [file supp_erv509_supplementary_table_S1.pdf]

Table S1. Primers used in this study

Primer name	Target gene	NCBI accession no.	Forward-primer sequence(5'-3')	Reverse-primer sequence(5'-3')
LpPPH-F/-R	*LpPPH*	KT345726	TGAAGAGGTTATTGGTGAAC	TGAGTGGTCAGCATATACTTG
*LpPPH-5'*RACE	*LpPPH*	KT345726	--	CTTGGAGAGCCGCATTGACGTCAC
LpPPH-3'RACE	*LpPPH*	KT345726	GGGCTGGGACATTTCCTCTTCCA	--
LpPPH-CDSF/-CDSR	*LpPPH*	KT345726	atcaggaattcATGGAAGTGGTTTCCTCCAG	aaccgtcgacAGACACTACCCGTATGTTGGAG
AtPPH-CDSF/-CDSR	*AtPPH*	AT5G13800.1	atcaggaattcATGGAGATAATCTCACTGAACG	aaccgtcgacTGCAGACTTCCCTCCAAAC
LpPPH-qRTF/-qRTR	*LpPPH*	KT345726	ACCCAGGTGATTCAGGAAAG	CCTGACCTCACCAACCTTCT
LpPPH-RTF/-RTR	*LpPPH*	KT345726	ATGGAAGTGGTTTCCTCCAG	AGACACTACCCGTATGTTGGAG
AtPPH-RTF/-RTR	*AtPPH*	AT5G13800.1	ATGGAGATAATCTCACTGAACG	TGCAGACTTCCCTCCAAAC
LpeIF4A-qRTF/-qRTR	*LpeIF4A*	G0924770	AACTCAACTTGAAGTGTTGGAGTG	AGATCTGGTCCTGGAAAGAATATG
LpTEF1-qRTF/-qRTR	*LpTEF1*	GR522099	CGTGTGATCGAGAGGTTTGA	CGAATTTCCAGAGGGCAATA
Bar-F/-R	*Bar gene* in pEarlyGate103	--	TCAAGGCCAAGAGAAGGTCT	TGTGTGGGTGTGAATGTGAG
GFP-F/-R	*GFP gene* in pEarlyGate103	--	CTTCACTGCAAAGTCCCAAA	GAAGTTGGGCTGCCTTGTAT
SALK_000095-RP/-LP	T-DNA confirmation	--	TGTACAGGTTATCGGTGAGCC	CTACCAATCCTGGACTCCTCC
LBb1.3	T-DNA confirmation	--	ATTTTGCCGATTTCGGAAC	--
